# Ultrasound-Assisted Osmotic Dehydration of Apples in Xylitol Solution: Effects on Kinetics, Physicochemical Properties and Antioxidant Activity

**DOI:** 10.3390/molecules30112304

**Published:** 2025-05-24

**Authors:** Angelika Wojtyś, Sławomir Pietrzyk, Karolina Grzesińska, Robert Witkowicz

**Affiliations:** 1 Department of Food Analysis and Evaluation of Food Quality, Faculty of Food Technology, University of Agriculture in Krakow, Balicka Street 122, 30-149 Krakow, Poland; angelika.wojtys@urk.edu.pl (A.W.); karolina.grzesinska@student.urk.edu.pl (K.G.); 2 Department of Agroecology and Crop Production, University of Agriculture in Krakow, Mickiewicza Street 21, 31-120 Krakow, Poland; robert.witkowicz@urk.edu.pl

**Keywords:** osmotic dehydration, xylitol solution, ultrasound, antioxidant properties

## Abstract

In the present study, the effects of varying ultrasound treatment durations (5, 15, 30, and 45 min) applied prior to osmotic dehydration in xylitol solutions on apple tissues were investigated. The efficiency of the osmotic dehydration process was assessed by analyzing its kinetic parameters. In selected samples of osmotically dehydrated fruits, physicochemical properties were evaluated, including dry matter content, total acidity, pH, sugar profile, color attributes, total phenolic content, antioxidant activity (measured by DPPH and ABTS assays), and vitamin C content. Additionally, principal component analysis (PCA) was conducted to explore the relationships among the measured variables and to identify underlying patterns within the dataset. Osmotic dehydration in xylitol significantly modified the physicochemical and antioxidant properties of apples, promoting substantial water loss and partial replacement of natural sugars with xylitol. The results showed that ultrasound pretreatment markedly influenced these effects, with treatment duration playing a critical role. Shorter ultrasound applications (15–30 min) enhanced xylitol uptake while better preserving antioxidant activity and color, whereas longer ultrasound treatments (45 min) achieved greater mass transfer but led to higher losses of bioactive compounds compared to untreated samples.

## 1. Introduction

Osmotic dehydration (OD) is a widely recognized method in food processing, designed to enhance product quality and extend the shelf life of fruits and vegetables. This process, which involves immersing plant tissues in hypertonic solutions, facilitates selective water removal and solute uptake, thereby concentrating flavors and preserving key nutritional and sensory attributes—such as color, texture, taste, and aroma—similar to those of fresh fruits [1,2]. OD process is mainly employed as a pretreatment prior to other technological processes, such as hot-air frying, freeze-drying, or freezing [3]. Among various osmotic agents, sucrose remains the most commonly employed in fruit processing due to its wide availability, high efficacy, and cost-effectiveness. Numerous studies have investigated the application of sucrose solutions in the osmotic dehydration of different fruits, such as mango [4], apricot, chokeberry [5], kiwifruit [6], cranberry [7], and pear [8].

However, the growing awareness of diet-related health issues, such as obesity and diabetes, has prompted the search for novel osmotic agents with additional functional benefits. In this context, polyols, also known as sugar alcohols, have emerged as attractive alternatives. These compounds are low-calorie sweeteners characterized by strong dehydrating properties and a sweetness intensity ranging from 30% to 100% relative to sucrose. Polyols demonstrate also anti-cariogenic, prebiotic, antioxidant, and antibacterial properties, while exerting minimal effects on blood glucose levels. Although no specific acceptable daily intake (ADI) has been established, excessive consumption may induce laxative effects due to incomplete intestinal absorption According to European Union legislation, seven polyols are classified as nutritive sweeteners: sorbitol, mannitol, isomalt, maltitol, lactitol, xylitol, and erythritol [9,10,11].

Parallel to the search for healthier osmotic agents, advances in processing technologies have introduced ultrasound—a mechanical wave characterized by sound frequencies from 20 kHz to 100 MHz. In food science, ultrasound serves as a non-thermal processing technique capable of inducing distinct physical and chemical modifications in food matrices. Overall, ultrasound generates rapid cycles of compression and decompression within the material, leading to the so-called “sponge effect”. This phenomenon promotes the formation of microchannels within the food structure, significantly enhancing mass transfer processes such as water loss and solid gain during dehydration. Moreover, ultrasound application can trigger cavitation—the formation, growth, and collapse of microbubbles—which produces localized high temperatures and pressures, leading to the formation of microchannels and structural modifications within the food matrix [12,13,14]. Numerous studies have demonstrated that the use of ultrasound, either before or during osmotic dehydration, significantly improves process efficiency. Fernandes et al. [15,16] reported that ultrasound pretreatment of pineapple and melon tissues led to notable structural breakdowns, resulting in higher water loss and sugar gain during osmotic dehydration in sucrose solution. Positive effects of ultrasound on mass exchange were also confirmed for kiwifruit by Nowacka et al. [3] and Prithani and Dash [17], who observed improved water and solute diffusivity following ultrasound pretreatment. Additionally, ultrasound-assisted osmotic dehydration in various solutions (sucrose, glucose, sodium chloride, maltodextrin) has been shown to better preserve product quality, including color, texture, and the retention of bioactive compounds such as antioxidants [18,19,20]. The effectiveness of ultrasound treatment depends on several operational parameters, including frequency, intensity, and duration, allowing precise tailoring of the process to optimize the texture, color, and nutritional value of food products. It is critical to carefully manage the ultrasound parameters to avoid excessive heating and texture degradation, often achieved by controlling the intensity or exposure time and/or utilizing cooling systems during processing [21].

The integration of ultrasound-assisted osmotic dehydration with the use of polyol-based solutions, particularly xylitol, represents a promising and synergistic strategy in modern food processing. By combining the advantages of accelerated mass transfer, improved nutritional composition, and reduced processing times, this approach offers a promising strategy for efficient and high-quality preservation in food processing.

However, a comprehensive understanding of the interactions between ultrasound parameters, xylitol concentration, and product quality attributes remains limited. Therefore, the aim of this study was to investigate the effects of different ultrasound treatment durations applied prior to osmotic dehydration in xylitol solution on fruit tissues, with particular emphasis on antioxidant activity, mass transfer efficiency, and physicochemical quality attributes.

## 2. Results and Discussion

### 2.1. Kinetic Parameters of the Osmotic Dehydration Process

Osmotic dehydration is a bidirectional mass transfer process in which water is removed from the material into a hypertonic solution, while solutes from the osmotic medium diffuse into the material due to the osmotic potential gradient [22]. To assess mass transfer during the osmotic dehydration of apples, the following parameters were analyzed: dry matter content (DM), water loss (WL), mass loss (ML), and solid gain (SG).

The performed two-factor analysis demonstrated the independent effect of both investigated factors, TUT (time of ultrasound treatment) and TOD (time of osmotic dehydration), on the osmotic dehydration process of apples (Table 1). Evaluating the impact of TOD, it can be unequivocally stated that an increase in dehydration time resulted in higher values of the analyzed parameters (WL, ML, SG, and DM). For three parameters (WL, SG, and DM), six homogeneous groups were identified, indicating that each 30 min increase in dehydration time led to a statistically significant rise in these parameter values. For the MS parameter, four homogeneous groups were identified, including three multi-element groups. The evaluation of the impact of TUT on the osmotic dehydration process of apples is not as straightforward. An increase in TUT values leads to a systematic rise in the analyzed parameters, with a slight irregularity observed for the MS parameter. However, the values of the analyzed parameters for the control sample do not always exhibit the lowest values, except for the DM parameter.

**Figure 1:**
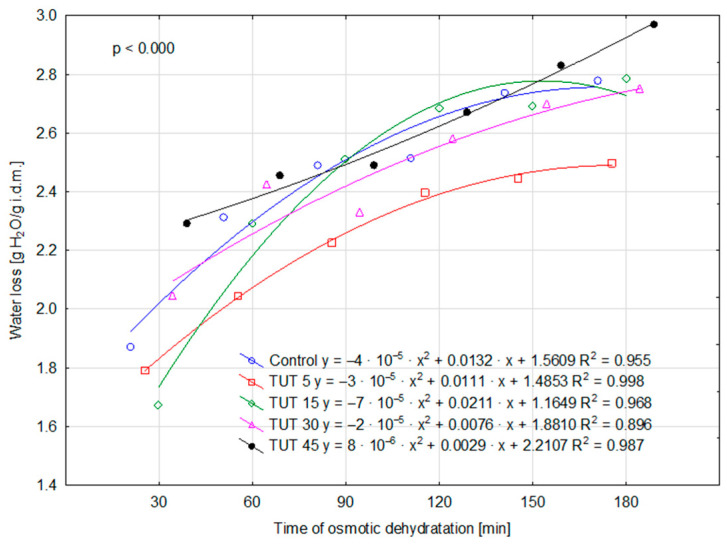
Variability of WL under the influence of the TUT × TOD interaction. Abbreviations explained in Table 1.

**Figure 2:**
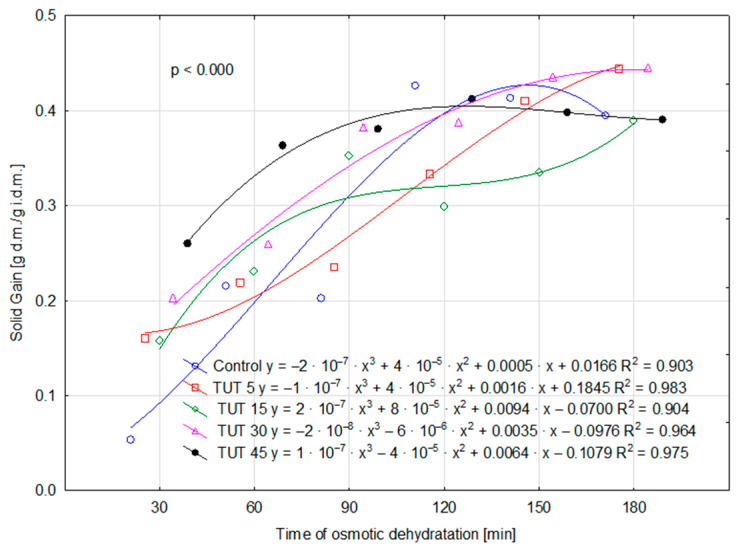
Variability of SG under the influence of the TUT × TOD interaction. Abbreviations explained in Table 1.

**Figure 3:**
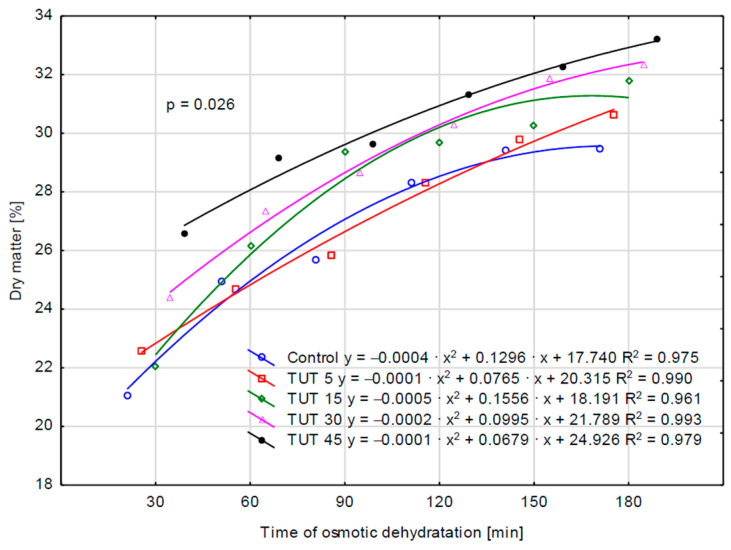
Variability of DM under the influence of the TUT × TOD interaction. Abbreviations explained in Table 1.

For the remaining parameters (WL, ML, and SG), the values determined for the control sample are higher—although not always statistically higher (SG parameter)—than those observed for the TUT 5 sample. This may suggest that a 5 min ultrasound treatment of the fruit resulted in a reduction in WL, MS, and SG values compared to those observed in the control sample. Therefore, it can be hypothesized that such ultrasonic treatment (TUT 5) may have led to an increase in cell wall impermeability, which, in turn, could be attributed to structural modifications primarily in cellulose and lignin. The performed analysis of variance also confirmed the statistical significance of the interaction effect between factors (TUT × TOD) for the parameters WL, SG, and DM (Figure 1, Figure 2, and Figure 3, respectively), as evidenced by the probability levels presented in the figures. Water loss over time was additionally illustrated using curves that account for the prior ultrasound treatment of the apples (Figure 1). Assessing the kinetics of this process, it can be observed that the shape of the four curves is similar, as they exhibit a negative polynomial slope coefficient. This indicates a deceleration in the dehydration process over time, both in cases where the fruits were not subjected to ultrasound treatment and those where they were treated for 5, 15, and 30 min. This decline is primarily attributed to structural changes in the tissue, as well as a decreasing osmotic pressure gradient between the dehydrated fruit and the surrounding osmotic solution over time [23]. The obtained findings are consistent with trends observed in other studies on osmotic dehydration processes. Brochier et al. [24], in their study on the osmotic dehydration of yacon roots in sorbitol, glycerol, and polydextrose solutions, also observed the highest rate of water loss at the beginning of the process. They emphasized that the increase in soluble solids occurred mainly within the first two hours of dehydration. Similarly, Wiktor et al. [9] reported a rapid initial water loss during the osmotic dehydration of organic strawberries in sucrose, sorbitol, and maltitol solutions. Cichowska et al. [13], on the other hand, found that during the osmotic dehydration of apples in 30% erythritol, xylitol, maltitol, and dihydroxyacetone solutions, solid migration into plant tissue continued even during the third hour of the process. However, according to their earlier research [25], particularly in erythritol and xylitol solutions, extending the process beyond three hours appears unnecessary, as further water loss in later stages does not differ significantly from that observed earlier. Ultrasound treatment of the fruit for 45 min altered the curve shape (positive polynomial slope coefficient), significantly increasing the rate of water loss over time. By the 180th minute of dehydration, this loss reached approximately 3 g H_2_O/g i.d.m. For the SG parameter, two polynomial functions had a positive slope coefficient (TUT 15 and TUT 45), while three exhibited a negative coefficient (control, TUT 5, TUT 30) (Figure 2). The TUT 45 curve indicates a decrease in SG values after exceeding 120 min of the dehydration process. Considering the shape of the WL parameter curve for this treatment (TUT 45), it can be concluded that such prolonged ultrasound exposure accelerates water loss, leading to an increase in DM content (Figure 3). However, the rate of dry matter accumulation (SG parameter) decreases. This may suggest a reduced rate of dry matter component replacement by xylitol.

**Figure 4:**
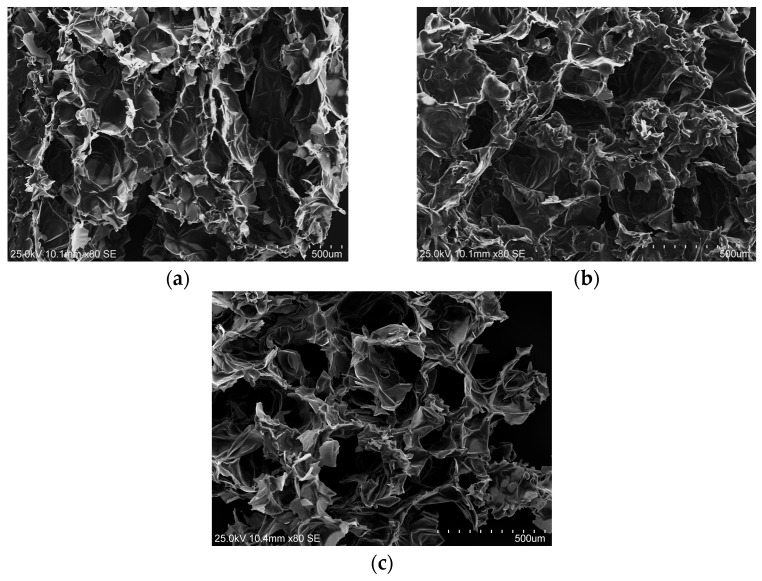
Micrograph SEM images (magnification 80×) of osmotically dehydrated apples: (**a**) control (TUT = 0), (**b**) TUT 5 + TOD 180, (**c**) TUT 45 + TOD 180.

Scanning electron microscopy (SEM) analysis (Figure 4a–c) showed the significant impact of ultrasound treatment on the tissues of the analyzed samples, directly correlating with the observed osmotic dehydration kinetics. Compared to the control sample (Figure 4a), the microstructure in the samples subjected to ultrasound treatment demonstrated a less ordered arrangement, with individual cells exhibiting a greater degree of mechanical damage. This phenome can be attributed to the cavitation effect generated by the ultrasonic waves. Samples subjected to 45 min of ultrasound (Figure 4c) exhibited substantial microstructure disruption and a highly enhanced porosity, compared to the control sample (characterized by a more homogeneous and compact tissue structure, exhibiting partially preserved cellular integrity with diminished intercellular spaces). In turn, samples subjected to 5 min of ultrasound treatment (Figure 4b) presented an intermediate degree of structural alteration. The increase in porosity and tissue degradation caused by ultrasound, particularly under prolonged exposure, appears to facilitate the initial stages of mass transfer by increasing the surface area and creating channels. Nevertheless, extended ultrasonic treatment may result in excessive structural degradation, potentially compromising cellular integrity and reducing effective permeability, which in turn may contribute to a diminished mass transfer rate during the later stages of the OD process. The observed structural alterations, along with a decreasing osmotic pressure gradient during dehydration, may constitute pivotal factors influencing the deceleration of dehydration kinetics over time.

Nowacka et al. [3] also demonstrated that ultrasound pretreatment longer than 10 min positively influenced mass exchange by enhancing water loss and solid gain. This was achieved through the creation of microchannels in the tissue structure. The authors confirmed this phenomenon using SEM and TD-NMR, which revealed structural changes in the tissue, including the formation of microchannels and slight redistribution of water within the cells. These alterations facilitated moisture removal and increased diffusivity. Such modifications, particularly when ultrasound treatment lasted for 20–30 min, were crucial in enhancing the osmotic dehydration process in kiwifruit. In the present study, ultrasound exposure for 45 min accelerated water loss but also resulted in a decrease in SG values after 120 min of dehydration, suggesting that prolonged sonication might have led to a reduced ability of the tissue to retain dry matter. These modifications likely increased the permeability of the cell wall, facilitating water removal but hindering the exchange of dry matter, which may explain the observed decrease in SG.

**Figure 5:**
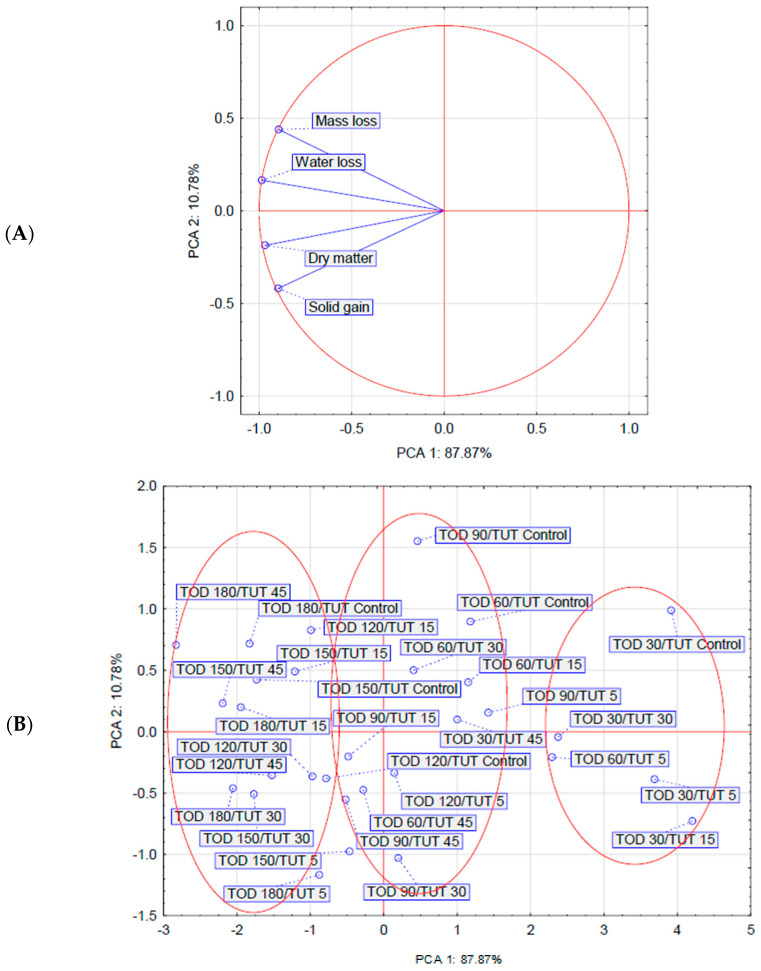
Biplot based on first two principal component axes for dehydration parameters of osmotically dehydrated fruits (**A**) and distribution of dehydration methods based on the first two components obtained from principal component analysis (**B**). Abbreviations explained in Table 1.

Based on the parameters analyzed above (WL, ML, SG, and DM), a principal component analysis (PCA) was performed, which demonstrated the variability of the dehydrated raw material. Although the first two components explain nearly 99% of the total variance, the first component alone accounts for almost 88% of the total variance. The analysis of the factor coordinates of the cases (Figure 5B) indicates that most of the variance explained by the first component is due to osmodehydration time. It can be observed that the values of all variables included in the analysis increase with the extension of the dehydration time. However, the factor coordinates of the cases do not allow for a definitive determination of the effect of ultrasound treatment on the analyzed parameters. The analysis also reveals correlations between ML and WL, as well as between DM and SG (Figure 5A).

### 2.2. Physicochemical and Antioxidant Properties of Osmotically Dehydrated Apples

#### 2.2.1. Effect of Osmotic Dehydration Process on Dry Matter Content, Water Activity, Total Acidity, pH, and Sugar Profile of Analyzed Apples

Fresh apples were characterized by a dry matter content of 14.73% (Table 2). This is consistent with results reported by Łata [26] and Ticha et al. [27], who reported that the dry matter content in fresh apples ranged from 13.6% to 19.3% and 12.4% to 20.0%, respectively, depending on the cultivar. The osmodehydration process in xylitol solution resulted in an 2-fold increase in dry matter content. An increase in dry matter content in osmotically dehydrated apples was also observed with longer ultrasound treatment durations. Higher values of dry matter in osmotically dehydrated apples compared to fresh fruits were mainly caused by the penetration of soluble solids from the osmotic solution into the plant tissue. In turn, extended sonication durations amplified this effect by accelerating mass transfer processes [2,11].

The water activity (a_w_) of fresh apples was 0.929. Following osmotic dehydration in a xylitol solution, a significant reduction in water activity was observed. The decrease in water activity can primarily be attributed to water loss, as well as the presence of hydroxyl groups in the xylitol molecules, which form hydrogen bonds with water molecules, thereby increasing the proportion of bound water in osmodehydrated fruits [13]

The control sample and samples treated with ultrasound for 5, 15, and 30 min showed similar water activity levels (ranging from 0.884 to 0.874), with no significant differences between them. In contrast, treatment with ultrasound for 45 min led to the lowest water activity value (0.840), indicating a significant reduction in free water content relative to the control and the shorter treatment times. Pearson’s correlation analysis demonstrated a strong negative correlation between aw values and both WL values (r = −0.877) and dry matter content (r = −0.907). The observed reduction in water activity can be explained by the mechanical effects of ultrasound, particularly cavitation, which induces the disruption of cell walls and increases the permeability of tissues. This facilitates water removal during osmotic dehydration, with prolonged ultrasound exposure leading to more profound structural changes and a more efficient reduction in available water. Nowacka et al. [7,28] also confirmed that 30 min ultrasound treatment does not affect the water activity of osmotically dehydrated kiwi and cranberry fruits, respectively.

The sweet and/or sour taste of apples is a key organoleptic characteristic that influences consumer satisfaction and consumption. The main compounds responsible for the characteristic sweet or sour taste of apples are primarily organic acids and sugars [29]. During the osmotic dehydration process, immersing the fruit in a hypertonic solution results in the simultaneous leaching of these soluble solids from the fruit tissue and the infusion of osmotic substances dissolved in the opposite direction, from the osmotic solution into the fruit tissue [12]. The total acidity and pH of the analyzed fresh apples were 0.82% and 3.79, respectively (Table 2). The osmotic dehydration process significantly reduced the total acidity of the fruits—by approximately fourfold—due to the exchange of substances between the fruit tissue and the osmotic solution, which led to a partial reduction in organic acids. Research conducted by Khoualdia et al. [30] and Fasogbon et al. [31], which investigated the osmotic dehydration of pomegranate peel and pineapple slices in sucrose solution, respectively, confirms the migration of acidic compounds from plant tissue into the osmotic solution during the process. Ultrasound treatment before osmotic dehydration further enhanced the loss of organic acids compared to the control sample. Regardless of the duration of ultrasound treatment, osmotically dehydrated apples exhibited significantly lower total acidity (up to approximately 80% lower) compared to the control sample. The total acidity values varied only slightly between different durations of ultrasound treatment. The higher pH values observed in osmotically dehydrated apples were primarily associated with the loss of organic acids during the process. The observed variations in acidity of analyzed samples may be attributed to the heterogeneous effects of ultrasound on the apple tissue microstructure. Ultrasound induces cavitation and cell wall disruption, which increases tissue permeability to water and soluble components during osmotic dehydration. Shorter sonication durations may primarily facilitate the migration of readily accessible acids from the tissue surface, whereas prolonged exposure may induce more extensive structural modifications within deeper tissue layers. As anticipated, a strong negative correlation was observed between the pH and total acidity of the analyzed samples (r = −0.978), indicating that higher pH values were associated with lower levels of total acidity.

Table 2 also presents the changes in the profile of selected sugars and the content of xylitol in the apples after the osmotic dehydration process (without and with US treatment). The sugar profile of fresh apples was primarily characterized by a fructose content of 43.09 g/100 g d.m. (dry matter) and a glucose content of 9.78 g/100 g d.m., which corresponds to 6.39 g and 1.45 g/100 g f.m. (fresh matter), respectively. These results are consistent with those reported by Ticha et al. [27]. The osmotic dehydration process caused significant changes in the sugar profile of the analyzed apples. Osmotic dehydration in the xylitol solution (without ultrasound) resulted in the loss of nearly 87% of fructose and 16% of glucose. The samples subjected to ultrasound treatment exhibited higher losses of simple sugars, with the extent of these losses increasing as the treatment duration progressed. Depending on the time of ultrasound treatment (5–45 min), the fructose content in osmotically dehydrated apples was reduced by 26–64%, while the glucose content decreased by 10–32% compared to the control sample. Statistical analysis revealed a strong negative correlation between both fructose and glucose content and the WL parameter (fructose: r = −0.992; glucose: r = −0.819). Furthermore, during osmotic dehydration, xylitol molecules diffused from the solution into the fruit tissue. The control sample was characterized by a xylitol content of 57.84 g/100 g d.m., while ultrasound treatment prior to the osmotic dehydration process, as expected, enhanced the diffusion of polyol molecules from the solution into the fruit tissue. The highest content of xylitol was found in osmodehydrated apples subjected to 30 min US treatment. The obtained results were about 15% higher than in control samples. In turn, the application of a longer US time resulted in a lower polyol content in osmotically dehydrated apples. According to Garcia-Noguera et al. [32], longer osmotic pretreatment times can cause breakdown of the fruit’s tissue structure, reducing resistance to molecular flow and decreasing the efficiency of sugar diffusion. It is consistent with the obtained SEM micrographs (Figure 4c), which revealed significant structural disintegration and increased porosity in apples subjected to 45 min of ultrasound treatment, indicating notable cell wall damage that may influence mass transfer dynamics. Pearson’s correlation analysis demonstrated a strong and significant correlation between the SG parameter and both xylitol (r = 0.998) and total sugar (r = 0.997) content. A significant replacement of natural sugars in plant tissue by polyol molecules during the osmotic dehydration process was also reported in our earlier study [33] and by Wiktor et al. [9]. Analyzing the sugar profile of osmotically dehydrated apples, it can be concluded that the use of a xylitol solution allows for the production of sweet-tasting products while significantly reducing total sugar content, thereby lowering the caloric value. The sweetness of xylitol is comparable to that of sucrose; however, its energy value is approximately 40% lower, providing 2.4 kcal/g compared to 4 kcal/g for sucrose [34,35]. Furthermore, xylitol is considered a metabolically favorable sweetener for individuals with diabetes due to its substantially lower glycemic index compared to sucrose. Specifically, the glycemic index of xylitol is 13, while those of sucrose, glucose, and fructose are 65, 100, and 23, respectively. This indicates that xylitol leads to a much slower and smaller postprandial increase in blood glucose levels. Its slow and partial absorption in the small intestine further contributes to this reduced glycemic response, making it a safe and effective alternative for individuals with both type 1 and type 2 diabetes [36,37]. It should also be noted that although no specific daily intake has been established for polyols (including xylitol), excessive consumption is known to induce gastrointestinal side effects, such as flatulence, abdominal cramps, laxation, and in severe cases, watery diarrhea—primarily due to their osmotic effects and fermentative degradation in the colon [38]. In accordance with European Commission Directive 94/54/EC, products containing more than 10% added polyols must carry the advisory statement “excessive consumption may produce laxative effects”, to ensure that consumers are properly informed. The tolerable daily intake of xylitol for adults is estimated to be approximately 100 g [39,40]. Additionally, the European Food Safety Authority (EFSA) [41] recommends that xylitol intake should not exceed 0.5 g per kg of body weight per day when consumed at spaced intervals, a level considered safe and unlike to cause adverse effects.

#### 2.2.2. Effect of Osmotic Dehydration Process on Total Phenolic Content Vitamin C Content and Antioxidant Activity in the Analyzed Apples

The total phenolic content (TPC) in fresh apples and those subjected to osmotic dehydration (without and with US treatment) in xylitol solutions is shown in Table 3. The TPC in the analyzed fresh fruits was 4.52 mg GAE/g d.m., which corresponds to 0.69 mg GAE/g f.m. As expected, the osmotic dehydration process, both without and with ultrasound pretreatment, caused a significant decrease in the total phenolic content of apples.

Yu et al. [42] investigated the total phenolic content in osmotically dehydrated blueberries as well as in the osmotic solution following the OD process. Their findings demonstrated that a considerable proportion of polyphenolic compounds (approximately 78%) diffuses from the fruit matrix into the surrounding liquid phase during osmotic dehydration. This mass transfer phenomenon leads to a reduction in polyphenol concentration within the dehydrated fruits and an accumulation of these bioactive compounds in the osmotic solution. In our study, a negative correlation was found between the WL parameter and total phenolic content (r = −0.840). The most pronounced reduction in TPC, approximately 60% compared to fresh fruits, was observed in apples subjected to 45 min of ultrasound treatment. In the case of other osmotically dehydrated samples, the decline ranged from 25% to almost 35%, although no statistically significant differences were observed between them. The greater loss of phenolic compounds observed in the TUT 45 + TOD 180 sample, compared to other treatments, may be attributed to more pronounced structural modifications in the fruit tissue induced by cavitation phenomena and microstreaming effects. These disruptions can compromise cellular integrity, facilitating the leaching of phenolic compounds, particularly due to the bidirectional mass transfer in the osmotic dehydration process [43].

Furthermore, prolonged ultrasound exposure may promote oxidative degradation and enhance enzymatic activity, accelerating the breakdown of phenolic compounds. During osmotic dehydration, fruits are immersed for an extended period at a temperature near the optimal range for polyphenol oxidase (40 °C), which further facilitates the degradation of phenolic compounds, including flavonoids and phenolic acids [44,45]. Differences in the content of phenolic compounds depending on the time of ultrasonic treatment were also noted by Pirce et al. [44]. In some raw materials, such as apples, plums, strawberries, and sour cherries, the presence of ultrasound during the osmotic dehydration process in various osmotic solutions has been noted to have either no significant effect or even a positive impact on phenolic content [14,18,46,47]. This suggests that changes in phenolic compound content are influenced by multiple factors, including the raw material, dehydration time, ultrasound treatment conditions, temperature, and the osmotic agent used.

Ascorbic acid is a highly labile compound that undergoes degradation when subjected to adverse environmental factors such as intense illumination, elevated pressure, high thermal conditions, and the presence of molecular oxygen [48]. The content of vitamin C in fresh apples was 0.52 mg/g d.m., corresponding to 0.08 mg/g f.m. (Table 3). Overall, the application osmotic dehydration process (without and with US) led to significant losses of vitamin C compared to the fresh raw material. The loss of vitamin C during the OD process is primarily attributed to its water solubility and subsequent leakage into the osmotic solutions [49]. A significant reduction in vitamin C content caused by the ultrasound-assisted OD process was also reported by Nowacka et al. [50] and Sakooei-Vayghan et al. [51] in cranberries and apricots, respectively. In our study, the greatest losses of vitamin C were observed after osmotic dehydration in xylitol solution, amounting to 93% in comparison to fresh fruit. The use of ultrasound at various exposure times also influenced the reduction in vitamin C content; however, no statistically significant differences were noted in comparison to the control sample. An exception was the TUT 30 + TOD 180 sample, which exhibited relatively lower losses of this compound (80%) compared to the other osmotically dehydrated apples. Ultrasound treatment can induce both mechanical and chemical effects that disrupt plant tissue in a non-uniform manner, depending on the duration and intensity of exposure. These irregularities in tissue structure may affect the way ultrasonic waves propagate through the material, leading to localized differences in structural and compositional changes. As a result, some regions of the tissue may better retain vitamin C, while others may undergo greater degradation. This uneven distribution of effects may explain the increased levels of vitamin C observed in apples after 30 min of ultrasound treatment [48,52]. Moreover, under specific conditions, ascorbic acid in food matrices can reversibly oxidize to L-dehydroascorbic acid through a redox reaction [53]. Extended exposure to ultrasound may facilitate the reverse reduction process, converting dehydroascorbic acid back into its reduced form. This mechanism may partially account for the slightly elevated ascorbic acid levels detected in apples subjected to 30 min of ultrasound treatment.

The osmotic dehydration process resulted in a significant decrease in the antioxidant activity (AA) of apples, as measured by both ABTS and DPPH assays (Table 3). In all analyzed fruits, the ABTS assay showed higher values than DPPH. Although both techniques used to assess antioxidant activity are based on electron transfer [9], the differences in the obtained results could be influenced by various mechanisms of scavenging of these two free radicals (such as reagent concentration, incubation time, or differences in reaction kinetics). The decrease in antioxidant activity after the OD process assisted by US is consistent with the results reported by Pirce et al. [44]. According to the authors, this reduction could be attributed to the migration of antioxidant compounds from the fruit to the liquid medium and the modifications occurring in the fruit tissues, which facilitate the transfer of components from the fruit to the liquid by decreasing resistance. In our study, Pearson’s correlation analysis revealed a strong negative correlation between the WL parameter and both DPPH (r = −0.843) and ABTS (r = −0.819) assays. The reduction in antioxidant activity (measured by both ABTS and DPPH assays) in the control sample was approximately 30% compared to fresh fruits. Similar to the total phenolic content, the most significant changes in the antioxidant activity of osmodehydrated apples were observed in those subjected to 45 min of ultrasound treatment, exhibiting approximately 35% lower antioxidant activity in both ABTS and DPPH assays compared to the control sample. This can be attributed to more significant structural changes in the fruit tissue induced by cavitation and microstreaming effects, which enhance the leaching of compounds with antioxidant properties. For shorter ultrasound treatment times, no statistically significant effect on the antioxidant activity of osmotically dehydrated fruits was observed. Pearson’s correlation analysis demonstrated a strong and significant correlation between the DPPH and ABTS assays (r = 0.916), as well as between total phenolic content and antioxidant activity as measured by both ABTS (r = 0.924) and DPPH (r = 0.955) assays.

#### 2.2.3. Effect of Osmotic Dehydration Process on Color Parameters of Apples

Color is one of the key quality parameters of fruit products and serves as the primary criterion for consumer quality assessment. Monitoring color changes during osmotic dehydration is of critical importance, as color degradation represents one of the most prominent quality alterations associated with this process. The obtained results of color parameters are presented in Table 4. The color of the fresh apple was characterized using the CIE Lab* color space, with values of L* = 73.74 (lightness), a* = 0.18 (redness), and b* = 22.36 (yellowness). In addition, derived color parameters—hue angle (h°), chroma (C*), and browning index (BI)—were also calculated. The corresponding values for the untreated sample were 22.37 for h°, 89.56 for C*, and 34.99 for BI. During the osmotic dehydration of apples in a xylitol solution, notable changes in color parameters were observed. A decrease in lightness was recorded across all processed samples compared to fresh apples, with the control sample (without ultrasound) showing the most pronounced reduction. This darkening is likely due to water loss and browning reactions—both enzymatic and non-enzymatic—as well as pigment degradation [54,55]. Ultrasound-assisted osmotic dehydration led to significantly smaller changes in color parameters, indicating a protective effect of ultrasound on color retention. The TUT 15 + TOD 180 sample showed higher lightness compared to the control, suggesting that optimal ultrasound pretreatment affects tissue brightness retention. Ultrasound treatment may increase L* values by promoting the deposition of sugars (including polyols) on the fruit surface [56], cavitation enhances cell membrane permeability, facilitating mass transfer and leading to sugar accumulation. This forms a translucent, light-reflective layer that boosts L* and may reduce browning by limiting oxygen access, further preserving lightness.

The increase in the a* parameter value across all treated samples could be affected by pigment concentration due to water loss. The increase in a* was positively correlated with ultrasound duration, reaching a peak in the TUT 45 + TOD 180 sample. In turn, the obtained b* parameter values revealed that the control sample exhibited the highest value, while ultrasound-treated samples displayed progressively lower values. This decrease suggests that ultrasound treatment might lessen excessive yellowing, potentially by limiting browning reactions or changing pigment composition. Pearson’s correlation analysis revealed a strong correlation between WL and both L* (r = −0.904) and a* (r = 0.984) parameters. Chroma, representing color saturation, followed a similar trend, with the control sample showing the highest saturation, and ultrasound-treated samples exhibiting more moderate values. The hue angle, describing the overall color tone, slightly decreased in the control and short-duration ultrasound samples, indicating a shift toward red tones. However, samples treated with ultrasound for 15 and 30 min showed hue values closer to those of fresh apples, suggesting better preservation of the natural color. The browning index increased significantly in all processed samples, with the control sample reaching the highest value, while ultrasound treatment notably reduced browning, especially in the TUT 15 + TOD 180 sample. The color of apples is strongly influenced by naturally occurring pigments, which can undergo oxidation during pretreatment. The primary factors accelerating their degradation include elevated temperature and exposure to oxygen. Enzymatic browning also plays a key role in color changes, as brown pigments form from previously colorless polyphenols, altering the optical properties of the fruit [14]. Enzymatic browning is primarily catalyzed by the enzyme polyphenol oxidase (PPO) which oxidizes phenolic compounds to quinones that polymerize into brown pigments. PPO is naturally present in plant tissues and becomes active upon cell damage, which increases contact between the enzyme and polyphenolic compounds. According to the literature [57,58,59,60], high-intensity (20–100 kHz) ultrasound treatment significantly contributes to PPO inactivation through cavitation effects, which generate localized high temperatures, shear forces, and free radicals. These factors disrupt the enzyme’s tertiary structure, leading to its denaturation and a substantial decrease in catalytic activity. Finally, the obtained total color difference (ΔE) values indicate that the color of osmotically dehydrated apples visibly differed from that of fresh fruits. The ΔE values ranged from 7.72 (TUT 15 + TOD 180) to 13.94 (control), meaning that the color difference between the analyzed samples would be noticeable to an observer as two distinct colors (ΔE > 5) [61]. Ultrasound-treated samples showed less noticeable color changes than the control sample. Many studies have confirmed the significant role of color in consumers’ perception and acceptance of fruit-based products. According to Pathare et al. [62], color differences with ΔE > 5 are considered easily perceptible by the human eye, while ΔE values closer to the fresh product are more likely to be accepted by consumer. Although all samples in our study exceeded this threshold, those subjected to ultrasound treatment—particularly TUT 15 + TOD 180 and TUT 30 + TOD 180—exhibited the smallest ΔE values, indicating a closer resemblance to the color of fresh apples. This is consistent with earlier research demonstrating that ultrasound processing can reduce enzymatic browning and pigment degradation, thus enhancing color stability in fruit tissues. As color is a key determinant of quality and consumer appeal in minimally processed fruits, maintaining a visual appearance similar to that of fresh produce represents a desirable quality attribute. Therefore, the reduced ΔE in ultrasound-treated samples may be considered an advantage both technologically and from a sensory perspective.

### 2.3. Principal Component Analysis

**Figure 6:**
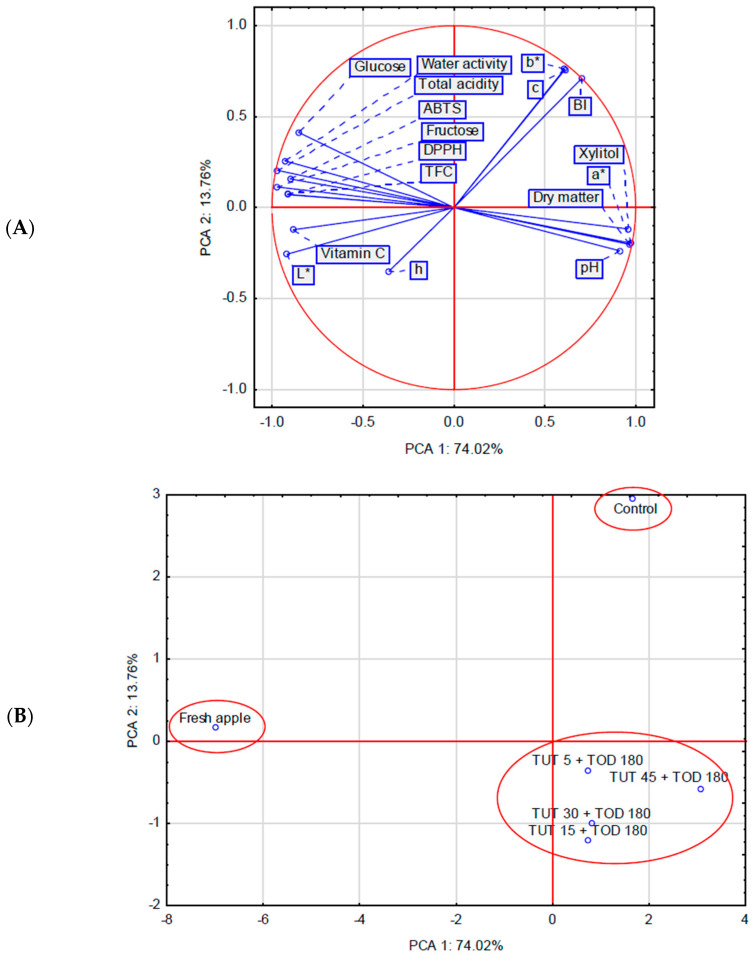
Biplot based on first two principal component axes for chemical and physical parameters of osmotically dehydrated fruits (**A**) and distribution of dehydration methods based on the first two components obtained from principal component analysis (**B**). Abbreviations explained in Table 1.

PCA confirmed a distinct influence of TUT (at a constant TOD = 180 min) on the physicochemical properties of the dehydrated raw material (Figure 6A,B). The 17 analyzed traits (TFC, DPPH, ABTS, total acidity, WL, DM, water activity, xylitol, glucose, fructose, vitamin C, and color parameters) included in the PCA allowed for the identification of three groups within the factor coordinate system, including two single-element groups ((1) fresh apple and (2) control) (Figure 6B). The first evident conclusion is the significant quality differentiation between fresh and dehydrated material. The osmotic dehydration process led to a reduction in the values of ten analyzed parameters, including TFC, ABTS, DPPH, vitamin C, and water activity. The control sample exhibited a pronounced decrease in L*, h, and vitamin C values, indicating a significant darkening and shift towards brownish hues. Simultaneously, certain color parameters, such as b*, C*, and BI, increased, reflecting an increase in yellow and color saturation, as well as an intensification of browning. These parameters were strongly negatively correlated with L* and h, further confirming the darkening and shift towards red and brown tones in the control samples. No significant correlations were observed between the color parameters b*, C*, and BI and the variables AA, pH, and dry matter content. The third group, located in the fourth quadrant of the coordinate system, consisted of samples in which the raw material was dehydrated using a xylitol solution (TOD 180 min) but had been previously treated with ultrasound (TUT 5-45). This resulted in a significant increase in dry matter content, xylitol concentration, pH, and the a* color parameter in the osmotically dehydrated material (linked to the concentration of red pigments as water is removed from the tissue). The remaining parameters exhibited either a slight (color parameters) or more pronounced (antioxidant activity) decrease. It was also observed that the dry matter content and pH of the osmotically dehydrated samples are negatively correlated with AA and TPC.

## 3. Materials and Methods

### 3.1. Chemicals

Sodium carbonate and 2,6-dichlorophenolindophenol (DCPIP) were purchased from POCh (Gliwice, Poland). Ethanol, sodium hydroxide, ascorbic acid, oxalic acid, sucrose, glucose, and fructose were obtained from Chempur (Piekary Śląskie, Poland). Acetonitrile, Folin–Ciocalteu reagent, 2,2-diphenyl-1-picrylhyrazyl radical (DPPH), 2,2′-azino-bis(3-ethylbenzothiazoline-6-sulfonic acid (ABTS), gallic acid, and 6-hydroxy-2,5,7,8-tetramethylchroman-2-carboxylic acid (Trolox) were purchased from Sigma-Aldrich Chemie (Steinheim, Germany).

### 3.2. Materials

Fresh apples of the Ligol variety were purchased from a local shop in Krakow, Poland. The fruits were stored under refrigerated conditions (6 ± 1 °C) until the analyses were conducted. Before the osmotic dehydration process, the apples were washed, peeled, cored, and cut into 2 × 2 × 2 cm cubes.

### 3.3. Ultrasonic Pretreatment Osmotic Dehydration Procedure

Osmotic dehydration was carried out according to the procedure of [7], with some modifications. The process conditions—the temperature and solution concentration—were selected based on our earlier studies [33], in which these parameters provided the most efficient mass transfer. The fruits were placed in beakers containing a 40% (*m*/*m*) aqueous osmotic solution of xylitol (Intenson, Karczew, Poland). The weight ratio of the osmotic medium to the samples was 4:1 to prevent changes in the solution concentration. Ultrasonic pretreatment was performed at 40 °C in an ultrasonic bath (Sonic 14, PolSonic, Warsaw, Poland) without mechanical agitation, using a frequency of 21 kHz and a total power of 400 W generated by sonotrodes, corresponding to an intensity of 8 W/g of fruits. During treatment, the fruits were covered with a net to prevent them from floating to the surface. The osmotic dehydration process was conducted over a total duration of 180 min, with the fruits subjected to ultrasonic treatment for 5, 15, 30, and 45 min. After US treatment, the samples were transferred to a shaking water bath (120 rpm), to continue the osmotic dehydration for the remaining time at a temperature of 40 °C. To evaluate the impact of ultrasound treatment, a control procedure without ultrasound was also conducted.

### 3.4. Kinetics Parameters

To determine the kinetic parameters of the osmotic dehydration process, the water loss, mass loss, and solid gain parameters were evaluated. The procedure was based on the method outlined by Kowalska et al. [63], with some modifications. The process was carried out for durations of 30, 60, 90, 120, 150, and 180 min in a water bath with agitation (210 cycles/min) at a temperature of 40 °C. After the osmotic treatment, the samples were removed from the solution, rinsed twice with distilled water, and dried on filter paper.

The kinetics parameters of the osmotic dehydration process were determined by calculating at the different time (τ) water loss, mass loss, and solid gain according to the following formulas [64]:

(1) $$WL=\frac{m_{0}\times {(100-dm}_{0})-m_{\tau }\times (100-{dm}_{\tau })}{m_{0}\times {dm}_{0}}\;[g\;H_{2}O/g\;i.d.m.]$$

(2) $$ML=\frac{m_{0}-m_{\tau }}{m_{0}}\times 100\;[\%]$$

(3) $$SG=\frac{m_{\tau }\times {dm}_{\tau }-m_{0}\times {dm}_{0}}{m_{0}\times {dm}_{0}}\;[g\;d.m./g\;i.d.m.]$$

where *m*_0_—initial mass of fresh sample; *m*_τ_—mass of sample after time τ of OD; *dm*_0_—initial dry matter of sample; *dm*_τ_—dry matter of sample after time τ of OD; i.d.m.—initial dry matter.

### 3.5. Scanning Electron Microscopy (SEM)

The microstructure of osmotically dehydrated apple samples was examined using a scanning electron microscope (SEM) (model S-3400N, Hitachi, Tokyo, Japan) operated at an accelerating voltage of 7 kV. For SEM analysis, small pieces were excised from the samples, mounted on aluminum stubs using double-sided conductive adhesive tape, and subsequently sputter-coated with a thin layer of gold using a 108/Auto sputter-coater (Cressington Scientific Instruments, Watford, UK). Micrographs were acquired at a magnification of 80×.

### 3.6. Determination of Physicochemical Properties

The dry matter content of fresh and osmotically dehydrated fruits was determined by oven drying: initially at 70 °C for 1 h, followed by drying at 105 °C until a constant weight was achieved. pH was determined using the potentiometric method. Total acidity (expressed as citric acid) was measured by titration with sodium hydroxide to the endpoint indicated by phenolphthalein [65]. Water activity was measured using a LabSwift-aw apparatus (Novasina AG, Lachen, Switzerland) in accordance with the manufacturer’s instructions at room temperature. All measurements were performed in triplicate.

### 3.7. Determination of Sugar Profile

Sugar extraction was carried out by immersing ground quince fruit in distilled water and subjecting it to ultrasonic treatment at 40 °C for 45 min. The resulting solution was clarified using Carrez reagents I and II, followed by filtration. The extracts were then purified through membrane filters (0.45 μm pore size) just before HPLC analysis. The sugar composition (fructose, glucose, sucrose, and xylitol) was quantified by high-performance liquid chromatography (HPLC) with refractive index detection (LaChrom, Merck, Hitachi, Japan), following the procedure outlined by Bogdanov et al. [66]. A mobile phase consisting of acetonitrile and water (80:20, *v*/*v*) was used, with a flow rate of 1 mL/min. The separation of the samples was performed on a Purospher Star NH2 column (Merck, Darmstadt, Germany) (250 × 4 mm, particle size 5 µm) with a pre-column at 30 °C. Individual sugars were identified by comparing the peaks in the sample with those of the corresponding standards.

### 3.8. Procedure of Ethanolic Extraction

Five grams of ground samples (both fresh and osmotically dehydrated) was extracted by shaking with 45 mL of 96% ethanol for 24 h. The resulting extracts were then filtered through filter paper and stored in a refrigerator at 6  ±  1 °C until further analysis. The extraction process was performed in triplicate.

### 3.9. Determination of Total Phenolic Content

The total phenolic content of ethanolic extracts from fresh and osmotically dehydrated apples was quantified using the Folin–Ciocalteu reagent, as described by Ainsworth and Gillespie [67]. Briefly, 0.1 mL of the extract was mixed with 2.5 mL of 0.2 M Folin–Ciocalteu reagent and allowed to react for 5 min. Subsequently, 2 mL of a 7.5% sodium carbonate solution was added, and the mixture was incubated at room temperature for 2 h to complete the reaction. The absorbance of the resulting solution was measured at 760 nm using a UV/VIS V-530 spectrophotometer (Jasco, Tokyo, Japan). TPC values were expressed as milligrams of gallic acid equivalents (GAE) per gram of dry matter of fruits.

### 3.10. Determination of DPPH Radical-Scavenging Activity

The DPPH radical-scavenging activity was evaluated following the method described by Larrauri et al. [68]. Briefly, 0.1 mL of ethanolic extract was combined with 3.9 mL of a DPPH solution (0.1 mM). The mixture was allowed to incubate for 15 min, after which the absorbance was recorded at 515 nm using a UV/VIS V-530 spectrophotometer (Jasco, Tokyo, Japan). The antioxidant activity against DPPH was expressed as µM of Trolox equivalents per gram of dry matter of fruits.

### 3.11. Determination of ABTS Cation Radical-Scavenging Activity

The ABTS cation radical-scavenging activity was assessed according to the methodology described by Baltrušaitytė et al. [69]. A 0.1 mL of the ethanolic extract was mixed with 6 mL of ABTS·+ solution and incubated for 30 min. Subsequently, the absorbance of the sample was measured spectrophotometrically at 734 nm. The antioxidant activity against ABTS was expressed as µM Trolox equivalents per gram of dry matter of fruits.

### 3.12. Determination of Vitamin C Content

The vitamin C content was quantified using the 2,6-dichlorophenolindophenol (DCPIP) titration method, in accordance with the AOAC official method [65]. The vitamin C content was expressed as mg of ascorbic acid per gram of dry matter of fruits.

### 3.13. Color Parameters

The color parameters of the samples were determined using a Color i5 spectrophotometer (X-Rite, Grand Rapids, MI, USA). The measurements were conducted under the following conditions: measuring geometry d/8°, illuminant D65, and a 10° observer angle. The equipment was calibrated using a white and black standard ceramic tile. The results were expressed in the CIE Lab* color space, where L* denotes lightness, a* represents the red-green axis, and b* corresponds to the yellow-blue axis. The chromatic attribute chroma [C* = (a*^2^ + b*^2^)^1/2^] and hue angle [h = tan^−1^(b*/a*)] were also determined [70]. The total color differences (ΔE) were computed using the formula described by Wojdyło et al. [71]:

$$\Delta E=\sqrt{{\left(\Delta L^{*}\right)}^{2}+{\left(\Delta a^{*}\right)}^{2}+{{(\Delta b}^{*})}^{2}}$$

where ΔL*, Δa*, and Δb* represent the differences in the mean L*, a*, and b* parameters, respectively, between fresh and osmodehydrated fruit.

Additionally, the browning index (BI) of the apples was determined using the formulas proposed by Buera et al. [72], as follows:

$$BI=\frac{100(X-0.31)}{0.172}$$

$$X=\frac{a^{*}+1.75L^{*}}{5.645L^{*}+a^{*}-3.012b^{*}}$$

### 3.14. Statistical Analysis

Two-factorial analysis of variance (ANOVA) and principal component analysis (PCA) were carried out using the STATISTICA v13.30 software (TIBCO Software Inc., Palo Alto, CA, USA). Tukey’s Honestly Significant Difference (HSD) at *p* = 0.05 was used to find the differences between means. The means denoted by different letters differed statistically. The kinetics of the osmotic dehydration process were described using the simplest possible polynomial models with a high determination coefficient. The kinetics of the process were described based on the time-dependent behavior of selected parameters, such as WL, ML, SG, and DM. In the PCA analysis, measurable variables were used, and all measurements were performed in triplicate. These variables included physicochemical properties characterizing both fresh apples and apples subjected to osmotic dehydration under varying conditions: (1) the presence or absence of ultrasound treatment (TUT); (2) different durations of osmotic dehydration (TOD). The analysis of the characteristics of the osmotically dehydrated samples included the following: (TFC, DPPH, ABTS, total acidity, WL, DM, water activity, xylitol, glucose, fructose, vitamin C, and color parameters).

## 4. Conclusions

This study investigated the impact of ultrasound pretreatment (5–45 min) on the osmotic dehydration of apples in a xylitol solution. Osmotic dehydration in xylitol significantly alters the physicochemical and antioxidant properties of apples, inducing substantial water loss and partial replacement of natural sugars (fructose and glucose) with xylitol. The obtained results suggest that ultrasound pretreatment significantly influences the effects of osmotic dehydration on apples, with the duration of ultrasound treatment being a critical factor in determining the final product’s characteristics. Longer osmotic dehydration times lead to increased water loss, mass loss, solid gain, and dry matter content, whereas the effect of ultrasound pretreatment is more complex and dependent on treatment duration. Generally, the obtained results indicate that shorter ultrasound treatments (15 and 30 min) are particularly effective in enhancing the absorption of xylitol, while simultaneously better preserving the antioxidant properties and color of the osmotically dehydrated apples. Extending the ultrasound treatment to 45 min results in the greatest mass exchange but also leads to higher losses of bioactive compounds compared to the control sample (without ultrasound). In conclusion, optimizing the duration of ultrasound treatment is essential for achieving a balance between enhanced mass transfer and preservation of the nutritional and sensory attributes in the final product. Future research should focus on determining the optimal ultrasound parameters to maximize xylitol absorption while minimizing the degradation of bioactive compounds, thereby contributing to the development of dehydrated apple products with improved health benefits (low-calorie and with a low glycemic index) and enhanced sensory appeal.

## Figures and Tables

**Table 1:** Kinetics of osmotic dehydration process of apples.

Factor	Factor Level	Water Loss [g H_2_O_2_/g i.d.m.] ^1^	Mass Loss [%]	Solid Gain [g d. m./g i.d.m.]	Dry Matter [%]
Time of ultrasound treatment (TUT) [min]	5 (TUT 5)	2.232 a	28.563 a	0.300 a	26.973 a
	15 (TUT 15)	2.438 b	31.702 b	0.294 a	28.216 b
	30 (TUT 30)	2.470 b	31.318 ab	0.352 b	29.175 c
	45 (TUT 45)	2.617 c	33.257 b	0.367 b	30.370 d
	Control (TUT 0)	2.449 b	33.463 b	0.284 a	26.481 a
Time of osmotic dehydration (TOD) [min]	30 (TOD 30)	1.933 a	26.148 a	0.167 a	23.343 a
	60 (TOD 60)	2.305 b	30.482 b	0.257 b	26.454 b
	90 (TOD 90)	2.408 c	31.216 bc	0.311 c	27.844 c
	120 (TOD 120)	2.568 d	32.884 bcd	0.372 d	29.603 d
	150 (TOD 150)	2.679 e	34.162 cd	0.398 d	30.716 e
	180 (TOD 180)	2.755 e	35.072 d	0.412 d	31.498 e

TUT 5—5 min of ultrasound treatment. TUT 45—45 min of ultrasound treatment. TOD 30—30 min of osmotic dehydration. TOD 180—180 min of osmotic dehydration. Control—sample without ultrasound treatment prior to osmotic dehydration. Within columns and factors, values subscribed by the same small letters did not differ significantly at *p* = 0.05. ^1^ i.d.m.—initial dry matter.

**Table 2:** Dry matter, water activity, total acidity, pH, and sugar profile of fresh and osmotically dehydrated apples.

Parameters	Fresh Apple	Control	TUT 5 + TOD 180	TUT 15 + TOD 180	TUT 30 + TOD 180	TUT 45 + TOD 180
Dry matter [%]	14.78 a	29.46 b	30.63 c	31.81 d	32.38 de	33.22 e
Water activity	0.929 c	0.884 b	0.875 b	0.874 b	0.875 b	0.840 a
Total acidity [%]	0.818 d	0.222 c	0.122 ab	0.118 ab	0.136 b	0.043 a
pH	3.79 a	4.62 b	4.86 d	4.89 d	4.77 c	4.74 c
Fructose [g/100 g d.m.]	43.09 e	5.65 d	4.18 c	3.44 b	3.16 b	2.00 a
Glucose [g/100 g d.m.]	9.78 e	8.16 d	7.34 c	7.42 c	6.55 b	5.56 a
Xylitol [g/100 g d.m.]	–	57.84 a	60.74 c	59.30 b	65.86 d	61.38 c
Total sugar [g/100 g d.m.]	52.87 a	71.65 d	72.26 d	70.16 c	75.57 e	68.94 b

Abbreviations explained in Table 1. Within rows, values denoted by the same small letters did not differ significantly at *p* = 0.05.

**Table 3:** The total phenolic content, vitamin C content, and antioxidant activity in fresh and osmotically dehydrated apples.

Parameters	Fresh Apple	Control	TUT 5 + TOD 180	TUT 15 + TOD 180	TUT 30 + TOD 180	TUT 45 + TOD 180
TFC [mg/g d.m.]	4.52 c	2.97 b	3.38 b	3.15 b	3.02 b	1.89 a
ABTS [μM TE/ g d.m.]	1543 d	1052 bc	1207 c	964 b	1052 bc	694 a
DPPH [μM TE/ g d.m.]	221 c	145 b	165 b	148 b	154 b	92 a
Vitamin C [mg/g d.m.]	0.52 c	0.04 a	0.04 a	0.05 ab	0.11 b	0.08 ab

Abbreviations explained in Table 1. Within rows, values denoted by the same small letters did not differ significantly at *p* = 0.05.

**Table 4:** Color parameters of fresh and osmotically dehydrated apples.

Parameters	Fresh Apple	Control	TUT 5 + TOD 180	TUT 15 + TOD 180	TUT 30 + TOD 180	TUT 45 + TOD 180
L*	73.74 f	62.67 a	63.49 b	66.41 e	66.17 d	64.93 c
A*	0.18 a	0.80 b	0.85 c	0.88 d	0.92 e	0.99 f
B*	22.36 a	30.30 e	24.11 b	24.25 b	25.15 c	25.99 d
C*	22.37 a	30.26 e	24.13 b	24.25 b	25.15 c	26.01 d
h°	89.56 c	88.55 b	87.99 a	90.14 d	90.41 d	87.82 a
BI	34.99 a	63.71 f	46.86 d	43.53 b	45.65 c	50.16 e
ΔE	–	13.94 e	10.59 d	7.72 a	8.37 b	9.60 c

Abbreviations explained in Table 1. Within rows, values denoted by the same small letters did not differ significantly at *p* = 0.05.

## Data Availability

The data presented in this study are available on request from the corresponding author.
